# Infliximab in a Child With Relapsing Tuberculous Meningoencephalitis: A Case Report and Literature Review

**DOI:** 10.1097/INF.0000000000005253

**Published:** 2026-05-04

**Authors:** Chiara Barone, Martina Capitani, Beatrice Fresch, Chiara Veredice, Marco Piastra, Paolo Frassanito, Roberta Onesimo, Danilo Buonsenso

**Affiliations:** From the * Area Pediatrica, Dipartimento di Scienze della Vita e Sanità Pubblica, Università Cattolica del Sacro Cuore, Roma, Italia; † Medicine and Sugery, Università Cattolica del Sacro Cuore, Roma, Italia; ‡ Child Neurology and Psychiatry Unit, Department of Neuroscience, Università Cattolica del Sacro Cuore, Fondazione Policlinico Universitario Agostino Gemelli IRCCS, Rome, Italy; § Paediatric Intensive Care Unit and Trauma Centre, Università Cattolica del Sacro Cuore, Fondazione Policlinico Universitario Agostino Gemelli IRCCS, Rome, Italy; ¶ Pediatric Neurosurgery, Fondazione Policlinico Universitario A. Gemelli IRCCS, Rome, Italy; ‖ Department of Woman and Child Health and Public Health, Fondazione Policlinico Universitario A. Gemelli IRCCS, Rome, Italy.

**Keywords:** infliximab, tuberculosis, meningitis, tuberculomas

## Abstract

A 5-year-old child with tuberculous meningoencephalitis developed severe drug-induced hepatitis after conventional antitubercular therapy. Consequently, standard treatment was discontinued, and second-line drugs were administered in combination with high-dose corticosteroids. However, the disease continued to progress significantly. The introduction of infliximab resulted in substantial clinical and radiologic improvement, highlighting its potential role in managing refractory cases of central nervous system tuberculosis.

Tuberculosis (TB) remains a significant global health concern, with an estimated 10.8 million new cases and 1.25 million deaths in 2023, making it one of the leading causes of infectious mortality worldwide.^1^ Children represent approximately 12% of all TB cases, with the highest incidence observed in those under 5 years of age: this group is particularly vulnerable to severe and disseminated forms of the disease, including tuberculous meningoencephalitis, which represents the most lethal form of extrapulmonary TB, with mortality rates ranging from 20% to 50%, despite appropriate antimicrobial therapy.^2,3^ A significant challenge in tuberculous meningoencephalitis management is the occurrence of paradoxical reactions (PRs), which are immune-mediated inflammatory responses that lead to worsening or new-onset neurological complications despite adequate anti-TB treatment.^4,5^ PRs have been reported in up to 30% of tuberculous meningoencephalitis cases, with associated morbidity and mortality due to increased intracranial pressure, infarctions, and hydrocephalus.^6^ Although corticosteroids are commonly used as the standard immunomodulatory treatment in tuberculous meningoencephalitis, a subset of patients continues to deteriorate, leading to increased interest in alternative therapeutic options, including tumor necrosis factor-alpha (TNF-α) inhibitors. Infliximab, a monoclonal antibody targeting TNF-α, has been explored as a potential adjunctive therapy for severe and steroid-refractory forms in tuberculous meningoencephalitis. A limited number of case reports describe its use in adults, but pediatric data are extremely limited.

## CASE REPORT

**FIGURE 1.**
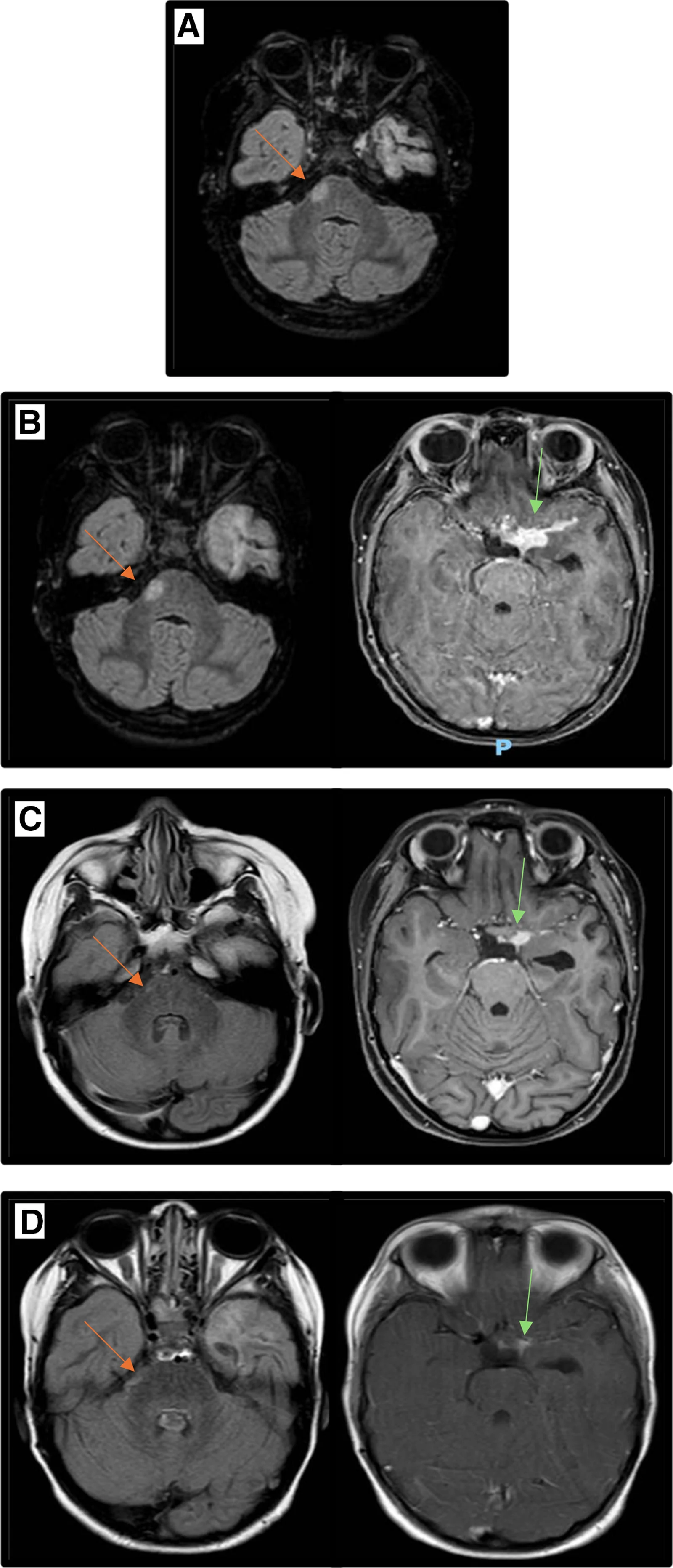
A: Transverse MRI brain sequences (axial 3D Flair) performed on day 60 (after 58 days of antitubercular therapy), showing gliotic-malacic evolution in both cerebral hemispheres, consistent with ischemic damage secondary to the initial vasculitic insult, along with the appearance of a contrast-enhancing nodule (<5 mm) protruding into the right prepontine cistern. B: Transverse MRI brain sequences (axial 3D Flair) performed at day 88 (after 72 days of nonconsecutive antitubercular therapy, discontinued for 14 days due to hepatotoxicity), showing stable gliotic-malacic changes in the caudate, putamen, thalamus, and nucleo-capsular regions, increased extent of edema in the left temporopolar and frontobasal cortico-subcortical regions, a slight increase in size (~5 mm) of the previously described contrast-enhancing nodule in the right prepontine cistern and the new appearance of a 7 mm ring-enhancing nodule in the left temporopolar region, consistent with a caseating granuloma (sT1W 3D Wats). C: Transverse MRI brain sequences, performed on day 110 (after administration of first and second dose of infliximab), showing a marked reduction in edematous changes in the left temporopolar and frontobasal cortico-subcortical regions, replaced by gliotic-malacic changes with initial signs of tissue loss; a reduction in size of the previously described ring-enhancing lesion adjacent to the basal cisterns (sT1W 3D Wats sequence, 5 mm vs. 7 mm in the previous report); and an almost complete resolution of the hyperintense T2/FLAIR lesion in the right lower pons. The previously enlarging nodular contrast-enhancing (CE) lesion in the adjacent cistern is now almost resolved, appearing as a pinpoint lesion. D: Transverse MRI brain sequences, performed on day 202 (3 months after discharge), showing continued regression of the infectious/inflammatory tissue within the left cisternal compartment. The previously reported round-shaped, T2/FLAIR hyperintense lesion with ring enhancement, is no longer appreciable. The punctate enhancement in the right pontomesencephalic cistern is resolved. MRI indicates magnetic resonance imaging.

A previously healthy 5-year-old boy was admitted to the hospital with a history of 15-day-persistent fever and pharyngodynia without cough, 2 months after returning from a trip to Bangladesh. At admission, the boy was alert, with a left deviation of the head and eyes, horizontal nystagmus and no focal deficits. Initial investigations showed leukocytosis with neutrophilia and mildly increased inflammatory markers (C-reactive protein 24.6 mg/L, procalcitonin negative) with a negative chest radiograph (CXR). Head-computed tomography scan revealed focal hypodensity in the left nucleo-capsular region, suspected for encephalitis, with dilation of the ventricular system. Two sets of blood cultures were performed—later found negative—and cerebrospinal fluid was collected for analysis: biochemical examination showed a pleocytosis (140 cells/mm^3^) and increased protein (223 mg/dL); molecular testing confirmed the presence of *Mycobacterium TB*, while culture remained negative after 6 weeks of incubation, precluding drug-susceptibility testing. A Quantiferon assay was also performed—returning a positive result—and brain magnetic resonance imaging (MRI) was obtained, showing findings consistent with tuberculous meningoencephalitis and revealing vasculitic involvement of the major vessels with consequent hemispheric infarcts and persistent supratentorial hydrocephalus. For this reason, he received isoniazid (H), rifampin (R), pyrazinamide (Z) and Ethambutol (E), along with oral corticosteroid therapy (dexamethasone) starting at 0.6 mg/kg/day (administered at day 2 and subsequently tapered until complete discontinuation after 8 weeks). The initial response was favorable, with remission of the reported headache and resolution of most of the neurological symptoms. On day 40, the patient developed recurrent episodes of vomiting, which showed poor response to prokinetic therapy and proton pump inhibitors. Due to persistent vomiting over several days, blood tests were performed, revealing significant hepatotoxicity on day 64, with elevated transaminase levels (alanine aminotransferasis at 2060 IU/L and aspartate aminotransferasis at 1436 IU/L). Liver function tests had been performed routinely and remained within normal limits until this point. Antitubercular treatment was therefore suspended, and only corticosteroid therapy was continued. Furthermore, a follow-up brain MRI was performed, showing disease progression with gliotic-malacic evolution in both cerebral hemispheres—indicating postinflammatory reparative and degenerative transformation of brain parenchyma and parenchymal loss—a worsening of the pathological tissue and the appearance of a new contrast-enhancing nodule protruding into the right prepontine cistern (Fig. 1A). Approximately 2 weeks later, following resolution of emesis and normalization of hepatic function, a gradual reintroduction of rifampin and isoniazid was attempted. Another follow-up MRI was performed 4 weeks after the previous scan, revealing an increase in size of the previously described nodule in the right prepontine cistern and a new appearance of a 7 mm ring-enhancing nodule in the left temporopolar region, consistent with a caseating granuloma (Fig. 1B). Consequently, given the high-risk localization of the growing tuberculomas despite steroid therapy, linezolid was added and an off-label treatment with infliximab intravenously was performed (5 mg/kg), with the first dose administered immediately, the second dose approximately 2 weeks later and the third 6 weeks after the first one. Sixteen days after reintroducing rifampin and isoniazid, the patient again developed elevated transaminases, prompting discontinuation of both drugs and initiation of levofloxacin on day 94, with subsequent progressive normalization of liver function tests. Dexamethasone was also reintroduced, again tapered and fully discontinued after 8 weeks. On day 111, rifampin was restarted as monotherapy, with serial monitoring confirming consistently normal transaminase levels. One month after the previous examination and the first dose of infliximab, another follow-up brain MRI was performed: it showed a marked reduction in infectious/inflammatory tissue, as well as a decrease in nodule size and surrounding edema and the resolution of the lesion involving the pontine region (Fig. 1C). Given the patient’s favorable clinical status and normal laboratory parameters while receiving rifampin, linezolid, levofloxacin and corticosteroid, discharge occurred on day 115. The patient was later readmitted for the third infliximab infusion (administered 6 weeks after the first dose, day 135): during this hospitalization, linezolid was discontinued due to the onset of neutropenia, while rifampin and levofloxacin were continued without complications. Three months after discharge, a follow-up brain MRI was performed, showing continued regression of the inflammatory tissue within the left cisternal compartment. The previously reported round-shaped T2-hyperintense lesion with ring enhancement was no longer appreciable, and the punctate enhancement in the right pontomesencephalic cistern had completely resolved (Fig. 1D).

After 12 months of follow-up, the patient remains on rifampin and levofloxacin alone, with normal liver function tests. From a clinical perspective, the child only has mild neurological deficits, such as horizontal nystagmus persisting, with mild intellectual disability (total IQ of 59 at the developmental evaluation Wechsler Preschool and Primary Scale of Intelligence—Third Edition), but is able to deambulate, talk and resume age-appropriate activities, regularly attending school.

**FIGURE 2.**
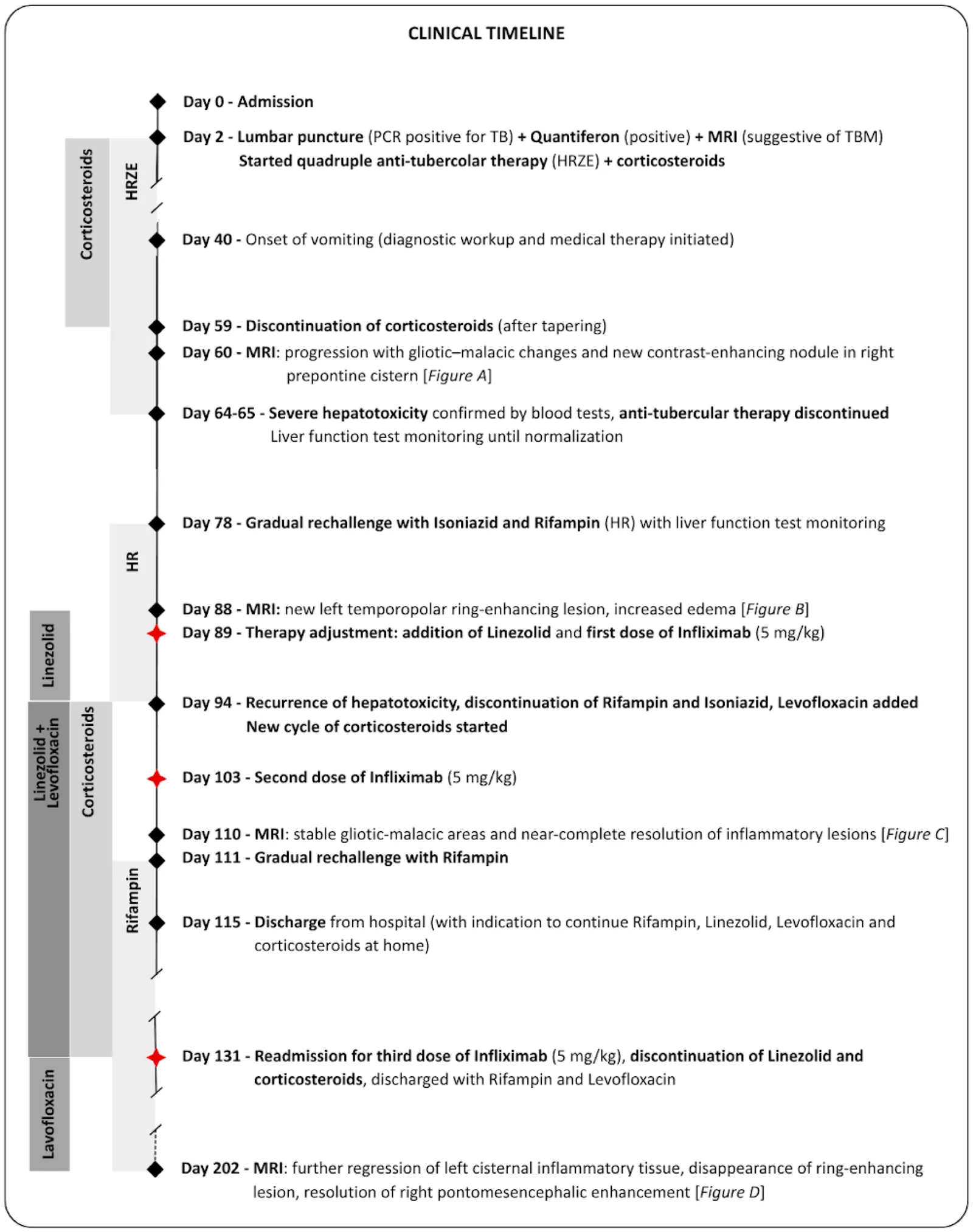
Timeline of clinical events: chronological sequence of clinical events, main exams performed, antitubercular therapy, corticosteroid cycles, infliximab administration and drug modifications due to hepatotoxicity.

The chronologic sequence of antitubercular and immunomodulatory therapies, corticosteroid cycles, and drug modifications due to hepatotoxicity is summarized in Figure 2.

## DISCUSSION

This case report highlights the efficacy and safety profile of infliximab in the management of central nervous system tuberculosis (CNS-TB), particularly in circumstances where first-line therapy proves ineffective or when standard treatments lead to significant adverse effects. In our patient, alternative treatment strategies were necessary, particularly given severe drug-induced symptomatic hepatitis, and, especially, the disease progression observed despite corticosteroid therapy. Infliximab was therefore introduced, after which a marked reduction in infectious and inflammatory tissue was observed, supporting its potential efficacy as an adjunctive option in the management of TB.

This therapeutic approach should be considered within the broader context of CNS-TB, a condition associated with substantial morbidity and mortality despite standardized antituberculous regimens and adjunctive corticosteroid therapy. CNS-TB remains a life-threatening disease, and among pediatric survivors, the majority experience neurological sequelae, emphasizing the severity of this condition and the urgent need for effective and targeted adjunctive strategies.^7–11^ In relation to the case presented, within the spectrum of therapeutic options potentially available for the management of CNN-TB, infliximab proved to be a central therapeutic strategy for our patient, who developed significant hepatotoxicity requiring discontinuation of conventional antituberculous therapy. Although its ability to cross the blood-brain barrier is limited, infliximab has demonstrated efficacy in reducing neuroinflammation in various conditions, even in TB meningitis (TBM) and its associated PRs. The rationale for using such treatment is based on the role of TNF-α—a cytokine that has been identified as an elevated marker in patients with TBM^12,13^—in the formation and maintenance of granulomas, essential immunological structures that localize and contain *Mycobacterium TB*. However, excessive TNF-α production has been implicated in tissue damage and in exaggerated inflammatory response, contributing to disease pathology.^14^ For this reason, corticosteroids are frequently employed as a first-line therapy in TB to attenuate inflammation, in part due to their capacity to suppress TNF-α and other pro-inflammatory cytokines. Nonetheless, prolonged corticosteroid therapy is associated with significant adverse effects, particularly in the setting of chronic or relapsing disease. Before infliximab was available, other corticosteroid-sparing treatments had also been used for refractory tuberculomas, such as thalidomide, and still with positive results.^15–17^ Following the use of thalidomide, another corticosteroid-sparing agent, infliximab, became available, and it has demonstrated an excellent safety and efficacy profile. We summarized all available cases of CNS-TB treated with infliximab or thalidomide in the available literature (by searching on PubMed “infliximab and tuberculosis” and “thalidomide and tuberculosis”) in Tables, Supplemental Digital Content 1 and 2, respectively https://links.lww.com/INF/G608 and https://links.lww.com/INF/G609, including both adults and children, to have a better comprehensive understanding on the potential benefits of this treatment. Tables, Supplemental Digital Content 1 and 2, https://links.lww.com/INF/G608 and https://links.lww.com/INF/G609 include approximately 180 patients treated with infliximab and 109 patients treated with thalidomide: among those receiving infliximab, approximately 34 were children and 146 adults, whereas thalidomide was administered predominantly in pediatric patients (approximately 92 children and 17 adults). In both cohorts, treatment was primarily reserved for severe CNS-TB, particularly in the setting of paradoxical neurologic deterioration or refractory disease despite optimized antituberculous therapy and corticosteroids, and when a corticosteroid-sparing strategy was required. Across reports, most patients experienced neurological improvement or stabilization. Focusing specifically on the use of infliximab, most of the available evidence comes from case reports, and there are currently no established guidelines recommending its routine use in TBM. The initial positive outcomes have been primarily documented in adult patients^18–20^; however, case reports also describe its successful use in pediatric cases. The first reports on the use of infliximab as adjunctive therapy in CNS-TB have emerged in recent years, for example in 2020 a young girl with optochiasmatic arachnoiditis, following TB treatment with adjunctive corticosteroids, was successfully treated with infliximab, leading to complete recovery^21^; another report from 2021 described 4 HIV-negative children who developed severe PRs unresponsive to corticosteroids,^22^ all of them showed clinical improvement after infliximab administration, reinforcing its safety and efficacy in such challenging cases.

In our patient, infliximab was introduced and administered in 3 doses, separated by a 2- and 4-week interval, at a dosage of 5 mg/kg (weeks 0, 2, and 6), a regimen previously described in pediatric case reports and primarily utilized in chronic inflammatory bowel disease. Considering that antitubercular therapy was interrupted and inconsistently administered due to repeated episodes of drug-induced hepatitis, it is highly likely that the observed reduction in tuberculoma burden was attributable to infliximab therapy rather than to anti-TB drugs. This finding is noteworthy as infliximab, widely used for inflammatory bowel disease, is typically associated with an increased risk of latent TB reactivation and therefore requires TB screening before initiation in that setting: in this case, however, infliximab was administered as an adjunct immunomodulatory therapy targeting excessive TNF-α-mediated inflammation resulting from TB itself, highlighting its potential role in managing severe or refractory cases.

In conclusion, this case report further supports the emerging role of TNF-α inhibition as an adjunctive therapeutic strategy in pediatric tuberculous meningoencephalitis: it may represent a targeted therapeutic option with the potential to become an increasingly valuable tool in the management of TB, not only for patients exhibiting deteriorating clinical and radiological parameters despite adequate antitubercular therapy and high-dose corticosteroids, but also for those experiencing significant adverse effects due to antitubercular drugs, requiring treatment interruption and developing disease progression, achieving excellent outcomes. However, further clinical studies are required to establish its efficacy and safety, particularly in the pediatric population.

## ACKNOWLEDGMENTS

*The authors thank the pediatricians, intensive care doctors, neurosurgeons and pediatric neurologists of Fondazione Policlinico Universitario A. Gemelli IRCCS, Rome, Italy, who have contributed to the comprehensive care of this patient.*

## Supplementary Material


