# Supplementary material for: Infliximab in a Child With Relapsing Tuberculous Meningoencephalitis: A Case Report and Literature Review
Source: Pediatr Infect Dis J. 2026 May 4;45(10):894–8. doi: 10.1097/INF.0000000000005253 (PMC13557465; doi:10.1097/INF.0000000000005253)
Supplement: Supplementary file 1 [file inf-45-0894-s001.pdf]

Supplemental Digital Content 1. Available literature on the use of infliximab in adults and children with Tuberculosis

Author,year	Age/Sex	Comorbidities	TB type	Treatment and length	Outcome
Yara- Natalie Abo, Dec 2021<br><br>*[Reference 22]*	11 m<br>F		Disseminated/miliary TB, TBM/CNS-TB, TB dactylitis.	Infliximab 5 mg/kg × 3 doses<br>0, 2 w, 6 w + dexamethasone 0.6 mg/kg/d (weaned over 6 w)	Normal development
	9 m<br>F		TBM/spinal TB	Infliximab 5 mg/kg × 3 doses<br>0, 2 w, 6 w + dexamethasone 0.6 mg/kg/d	Improving right hemiparesis and mild intellectual disability
	15 y<br>F		Disseminated/miliary TB, TBM/spinal TB	Infliximab 5 mg/kg × 3 doses<br>0, 2 w, 6 w + dexamethasone 0.4 mg/kg/d	Improved neurological symptom
	7 y<br>F		TBM	Infliximab 5 mg/kg ×3 doses<br>0, 2, 6 w + dexamethasone 0.6 mg/kg/d	Full neurological recovery
Ben J Marais<br>Dec 2020<br><br>[*Reference 23*]	36 y<br>M<br><br>India		Miliary TB, TBM, brain tuberculomas	dexamethasone (4–8 mg 3–4x/d) and/or oral prednisone (1–2 mg/kg/d - maximum 60 mg/d) for 3 m before infliximab and 4 m after Infliximab 10 mg/kg monthly x3	Mild cognitive deficit, assistance with activities of daily living
	32 y<br>F<br><br>China		Miliary TB, TBM, brain tuberculomas	steroids dexamethasone (4–8 mg 3–4x/d) and/or oral prednisone (1–2 mg/kg/d - maximum 60 mg/d) for 2 m before infliximab and 2 m after<br>Infliximab 5 mg/kg 0/2/6/14 weeks	Full neurological recovery
	55 y<br>F<br><br>Indonesia		TBM and necrotic lymphadenitis	steroids dexamethasone (4–8 mg 3–4x/d) and/or oral prednisone (1–2 mg/kg/d - maximum 60 mg/d) for 2 m before and 4 m after Infliximab 5 mg/kg monthly x3	Incomplete recovery with compromised sphincter function; regained mobility
	26 y<br>M<br><br>India		PTB with CNS and bone involvement. Multiple tuberculomas	steroids dexamethasone (4–8 mg 3–4x/d) and/or oral prednisone (1–2 mg/kg/d - maximum 60 mg/d) for 6 w before and 2 m after Infliximab 10 mg/kg, 0/3 weeks, 5 mg/kg, 7/17 wks	Regained sphincter function and mobility
James S Molton<br>Feb 2015<br><br>[*Reference 24]*	60 y<br>F		TBM	Dexamethasone + infliximab 10 mg/kg x 3 doses, 0/4/8 weeks	Mild cognitive deficit, assistance with activities of daily living
	32 y<br>F		CNS TB	Dexamethasone 12 mg/day + infliximab 5 mg/kg, x 3 doses (improved after 1 month)<br>Two doses of infliximab were given over the subsequent 6 weeks	Full neurological recovery but a seizure episode necessistated start of antiepileptic therapy
Timothy K. Blackmore<br>Nov 2008<br><br>[*Reference 25]*	42 y<br>F		Miliary TB	Dexamethasone 16 mg/day + infliximab 10 mg/kg x 3 doses, 0/4/8 weeks (12 months total)	Full neurological recovery, significant spasticity at lower limbs.
Denise C Hsu<br>Sep 2015<br><br>[*Reference 26]*	41 y<br>M	HIV	Miliary TB	Prednisone 80 mg/d + octerotide 50 microg 3xd + infliximab 4 mg/kg single dose	Clinically improved
	42 y<br>M	HIV	lymphnodal TB	Prednisone 75 mg/d + infliximab 5 mg/kg x 3 doses, 0/2/4 weeks	Lymphadenopathy was reduced
Grace Chia<br>Aug 2022	44 y<br>F		TBM with miliary lung involvement	Methylprednisolone 1g + infliximab 5 mg/kg (4 months)	Dead

[Reference 27]					
Abi Manesh<br>July 2023<br><br>*[Reference 28]*	Total (N=90)<br>Age (mean ± SD), y: 34.48 ± 11.8<br>Sex, male, n (%):54 (60)<br>HIV-positive status, n (%):7 (7.8)<br><br>Cohort A (n=30):<br>31.10 ± 8.7<br>15 male (50)<br>1 (3.3)<br><br>Cohort B (n=60):<br>36.17 ± 12.8<br>39 male (65)<br>6 (10)	HIV in 7 patients	TB meningitis (TBM), tuberculomas, tuberculous vasculitis, spinal involvement, and optochiasmatic TB	Cohort A (n=30): infliximab 10 mg/kg x3 doses, 0/4/8 weeks. Infliximab doses were stopped early if there was a complete response. The median duration from start of ATT and steroids to consideration of treatment with infliximab was 6 (IQR: 3.7–13) months.<br><br>Cohort B (n=60): treated with routine SOC (ATT and steroids). The median duration of ATT and steroids received was 12 (IQR: 7.3–18) months.	Cohort A was more likely to experienced disability-free survival and showed an higher likelihood of a successful outcome at 6 months compared with Cohort B (Relative risk, RR: 2.3; 95% confidence interval [CI]: 1.28–4.36). Additionally, severe disability (mRS score: 4 and 5) at 6 months was observed in 5 of 30 patients (16.7%) in cohort A compared with 21 of 60 patients (35%) in cohort B. Cohort A showed a trend towards reduced mortality compared with cohort B (2/30 [6.7%] and 13/60 [21.7%], respectively).
	38 y<br>M		Tuberculomas, abscesses, large vessel infarct, optochiasmatic arachnoiditis,hydrocephalus	High dose steroids (2 months) + infliximab 3, 10mg/Kg	Neurological conditions improved, mild slurred speech
	36 y<br>F		Cranial and spinal tuberculomas and optochiasmatic arachnoiditis	High dose steroids (7 months) + infliximab 3, 10mg/Kg (3 months)	Lower limb strength increased, no neurological deficits
	34 y M		Tuberculomas, infarcts	steroids (4 months) + infliximab 2, 10mg/Kg (2 months)	Residual memory deficits
	26 y<br>F		Optochiasmatic arachnoiditis, infarcts, hydrocephalus	steroids (8 months) + infliximab 3, 10mg/Kg (2 months)	Improved vision
	23 y<br>F		Tuberculoma, abscess, infarct obstructive hydrocephalus,	steroids (4 months) + infliximab 2, 10mg/Kg (2 months)	Seizures ceased, walks without support
	47 y<br>M		Tuberculomas, infarcts	steroids (7 months) + infliximab 2, 10mg/Kg (2 months)	Independent in all ADL
	19 y<br>M		Multiple Tuberculomas, infarcts, hydrocephalus	steroids (18 months) + infliximab 3, 10mg/Kg (1 month)	Complete recovery
	40 y<br>M		Intracranial spinal tuberculomas	steroids (6 months) + infliximab 3, 10mg/Kg (2 months)	Complete recovery
	30 y M		Tuberculomas	steroids (6 months) + infliximab 2, 10mg/Kg (1 month)	Complete recovery with intermittent amnesia
	39 y<br>F		Tuberculomas hydrocephalus, infarct	steroids (3 months) + infliximab 3, 10mg/Kg (3 months)	Residual right hemiparesis

	30 y M		Tuberculoma, hydrocephalus	steroids (5 months) + infliximab 3, 10mg/Kg (1 month)	Independent for all ADL, some emotional lability present
	24 y F		Tuberculomas, infarct	steroids (3 months) + infliximab 3, 10mg/Kg (1 month)	Independent to all ADL
	53 y M		Multiple tuberculomas	steroids (14 months) + infliximab 3, 10mg/Kg (3 months)	Full recovery
	22 y M		Multiple intracranial/spinal tuberculomas, hydrocephalus	steroids (7 months) + infliximab 2, 10mg/Kg (1 month)	Full recovery
	28 y F		Tuberculomas	steroids (13 months) + infliximab 3, 10mg/Kg (2 months)	Seizure free, walks well
	23 y F		Tuberculomas, hydrocephalus	steroids (6 months) + infliximab 3, 10mg/Kg (2 months)	Ataxia resolved
	18 y F		Multiple tuberculomas	steroids (6 months) + infliximab 3, 10mg/Kg (1 month)	Neurological recover
	22 y F		Cranial spinal tuberculomas	steroids (1 months) + infliximab 3, 10mg/Kg (3 months)	Walks independently
	29 y M		Spinal Arachnoiditis	steroids (18 months) + infliximab 1, 10mg/Kg (8 months)	Walks without support
	36 y F		Tuberculomas, infarcts	steroids (12 months) + infliximab 3, 10mg/Kg (12 months)	Walks independently, residual weak left-hand grip
	44 y M		Multiple tuberculomas, obstructive hydrocephalus	High dose steroids (7 months) + infliximab 3, 10mg/Kg (1 month)	Full recovery
	32 y M		Intra cranial and spinal tuberculomas	High dose steroids (6 months) + infliximab 3, 10mg/Kg (2 months)	Walks with AFO stick
	27 y M		Tuberculomas, obstructive hydrocephalus	High dose steroids (14 months) + infliximab 3, 10mg/Kg (1 month)	Residual cerebellar dysfunction present, hearing improved
	25 y M		Intracranial and spinal tuberculomas, hydrocephalus	High dose steroids (6 months) + infliximab 2, 10mg/Kg (2 months)	Mild improvement in upper limb power, neuropathic pain improved
	34 y F		Intracranial and spinal Tuberculoma	High dose steroids (14 months) + infliximab 3, 10mg/Kg (1 month)	Walks with support
	21 y F		Optochiasmatic arachnoiditis and infarcts	High dose steroids (3 months) + infliximab 3, 10mg/Kg	No improvement in vision, no motor or sensory weakness
	47 y F		Multiple infarcts in ICA and PCA territory	High dose steroids (1 months) + infliximab 1, 10mg/Kg	No response after the first dose
	33 y F		Cranial and spinal tuberculomas	High dose steroids (13 months) + infliximab 1, 10mg/Kg	No response after 1st infliximab dose- discontinued
	21 y M		Tuberculomas	High dose steroids (15 months) + infliximab 2, 10mg/Kg	Died after 5 months of last dose of infliximab
	32 y F		Cranial Spinal tuberculomas	High dose steroids (1 months) + infliximab 1, 10mg/Kg	Died 4 months after the last dose of infliximab
Jorge Jaimes-Hérnandez June 2012 *[Reference 29]*	20 y M	JIA (treated with infliximab)	Miliary tuberculosis, CNS TB, TBM. Multiple tuberculomas	Prednisone 75 mg/d+ infliximab 2.5 mg/kg and then 10 mg/kg single dose	No clinical reactivation of JIA and without neurologic symptoms
Miguel Santin Oct 2020 *[Reference 30]*	53 y F Dominican republic		Disseminated tuberculosis, CNS-TB, tuberculomas	dexamethasone 24 mg/day + infliximab 300 mg/kg x 3 doses, 0/2/6 weeks	Subarachnoid haemorrhage due to rupture of an aneurysm, but there was no evidence of active TB
	19 y M Pakistan		CNS-TB, tuberculomas	Dexamethasone 12 mg/day + infliximab 3 mg/kg x 6 doses	Residual deafness of the right ear
	Total (n=42) 13 adults 30 children		CNS Tb	2 patients (1 adult and 1 children) received corticosteroids as adjunctive treatment to anti-TB therapy. 13 adults were treated with infliximab 2.5 to 10 mg/kg (4 patients received 3 doses one month apart; 2 patients received 1 initial dose followed by 2 additional doses 2; 2 patients received 4 weeks apart; 1 patient received a single dose and the dose and duration were unknown), adalimumab 40 mg (1 patient 2 weeks apart and 1 patient 4 weeks apart)or thalidomide (27 patients received 1-24 mg/kg/day, 1 patient 100 mg/twice daily, 1 patient 100 mg/once daily. The duration of treatment was 2-9 months)	38 (83.5%) cases improved, of which 13 were adults and 25 were children. Although most children improved progressively within weeks to months after starting therapy, 2 children did not respond and could not ascertain the clinical response in another 2. 24 children (80.0%) survived and 56.7% experienced full recovery. Among adults, 11 (84.6%) were alive after treatment, but 53.8% of these were cured with sequelae of some type. One patient who initially responded died after 9 months from an intracerebral hemorrhage

Stefaan J. Vandecasteele<br>Sept 2012<br><br>*[Reference 31]*	58 y<br>M	HLA-B27 positive Spondylarthritis	TB lymphadenitis with lung lesions	Dexamethasone 12 mg + infliximab	Episode of vertigo 2 weeks after institution of infliximab. The BA stenosis progressively regressed
Aayesha J. Soni<br>Sept 2024<br><br>*[Reference 32]*	Total (n=19)<br>16 were adults (Median age: 32y);<br>3 were children (8,9 and 70 months)<br>10 male		All patients had non-bony manifestations of spinal TB (tuberculoma, epidural collections…), with two also having bony involvement (spondylodiskitis).	Corticosteroids were prescribed before TNF-α antagonist initiation in 14 patients. Decompressive spinal surgery was performed in four patients.<br>Three patients received thalidomide (doses of 2–5 mg/kg/day), 15 received infliximab(10 mg/kg x 1-3 doses) and one received both.	Seventeen (89.5 %) cases improved neurologically after TNF-α antagonist initiation, including all who received thalidomide (n = 4) and 14 out of 16 who received infliximab. At the end of monitoring, 15 patients could walk, and five had complete neurological recovery.
	21 y<br>F		CNS TB	Prednisone 2 mg/kg/day + infliximab 1 mg/kg/day	Full recover
Deborah Eshagh<br>June 2020<br><br>*[Reference 33]*	55 y<br>F		Multiple lymph nodes and osteolytic lesions. bilateral non-granulomatous anterior uveitis occurred.	Prednisone + Infliximab 5 mg/kg x 2 doses, 0/2 weeks. Infliximab was administered at 5 mg/kg in July, September, November 2019 and then every 4 weeks and dexamethasone and tropicamide eyedrops were given.	Biological remission
Richaud Clémence<br>June 2015<br><br>*[Reference 34]*	30 y<br>M<br>Senegal	HIV	Pleuropulmonary TB, abdominal adenopathies. TB-IRIS Steroid-dependent	High dose steroids + infliximab 5 mg/kg, x 6 doses monthly.	Regression
Myriam Briner<br>Aug 2021<br><br>*[Reference 35]*	36 y<br>F<br>Asian		Disseminated TB, followed by encephalitis	Dexamethasone 0.4 mg/kg/day (6 weeks), then 0.3 mg/kg/day (1 weeks) + infliximab 10 mg/kg x 2 doses, 0/4 weeks	Slight paraparesis and severe affection of the dorsal columns with impaired vibration sense
Yara-Natalie Abo<br>April 2020<br><br>*[Reference 21]*	7 y<br>F		Tuberculous meningitis with optochiasmatic arachnoiditis	Dexamethasone 0.15 mg/kg 8 hourly + infliximab 5 mg/kg x 3 doses, 0/2/6 weeks.<br>prednisolone 2 mg/kg/d for 2 weeks w slow weaning over 8 wks.	Full recovery
Dong Hyun Kim<br>Jan 2024<br><br>*[Reference 19]*	65 y<br>M		Tb meningitis	Dexamethasone + Infliximab 5-mg/kg After 6 weeks, a second course of infliximab 3 mg/kg.	Full recovery
Ryo Miyakawa<br>May 2024<br><br>*[Reference 36]*	16 y F<br>Latin America	COVID-19	Pulmonary TB, TBM with ocular involvement	Dexamethasone + infliximab 7.5 mg/kg/dose x 3 doses 0/2/6 weeks	Full neurological recovery
Lucas Armange<br>Feb 2023<br><br>*[Reference 37]*	33 y<br>F		Pulmonary TB, TB CNS	Prednisolone 5 mg/day + infliximab 10 mg/kg (6 months)	Mild neurological sequelae
	27 y<br>M		TB CNS	high-dose dexamethasone + infliximab 10 mg/kg x 3 doses monthly + prednisolone, 1 mg/kg/day (1 month)	At 3 years follow up, the patient had full recover
	26 y<br>F<br>Serbia	Pregnant (13th week)	Pulmonary TB, tuberculosis-related uveitis, granulomatous peritonitis	infliximab 5 mg/kg x 3 doses monthly	enlarged lymph nodes and the pre-sternal mass remained stable at 1 year, still asymptomatic
	61 y<br>F		Granulomatous abdominal collection	Dexamethasone 10 mg × 4/day + infliximab 5 mg/kg x 3 doses, 0/2/4 weeks	Improved

	Mayotte				
O'Dowd<br>2011<br><br>*[Reference 38]*	28 y<br>M	Crohn<br>(infliximab)	Lymph nodes	Prednisolone 40 mg/day + infliximab	Improved, no sequelae
