# Supplementary material for: Infliximab in a Child With Relapsing Tuberculous Meningoencephalitis: A Case Report and Literature Review
Source: Pediatr Infect Dis J. 2026 May 4;45(10):894–8. doi: 10.1097/INF.0000000000005253 (PMC13557465; doi:10.1097/INF.0000000000005253)
Supplement: Supplementary file 2 [file inf-45-0894-s002.pdf]

SUPPLEMENTAL DIGITAL CONTENT 2. Available literature on the use of Thalidomide in adults and children with Tuberculosis

Author, year	Age, sex	Comorbidities	TB type	Treatment and length	Outcome
Emanuela Caraffa<br>Jul 2018<br><br>*[Reference 15]*	9 y<br>F		TBM with tuberculomas. Tuberculous cerebral vasculitis	dexamethasone 16 mg/d + thalidomide 1.2 mg/kg/d (7 months)	Fully recovered
Ping Liu<br>Oct 2022<br><br>*[Reference 39]*	40 y F<br>China		TBM with pulmonary involvement	Dexamethasone + thalidomide 200mg (2 months)	Fully recovered
Patricia de la Riva<br>April 2013<br><br>*[Reference 17]*	35 y<br>F	35 week pregnant	Miliary TB with CNS involvement	Thalidomide 100 mg twice daily (8 months)	sensory polyneuropathy, which resolved. Three years after disease onset, the patient has a spastic gait but leads an independent life.
Johan F Schoeman<br>June 2010<br><br>*[Reference 16]*	6 y<br>M		Tuberculous meningitis with visual impairment	Prednisone+ Thalidomide 4.5 mg/kg/d (8 months)	Mild residual spastic diplegia, vision remained unchanged
	7 y<br>M	Acute lymphoblastic leukemia	Pulmonary TB, TBM	Prednisone 2 mg/kg/d+ Thalidomide 4.5 mg/kg/d (3 m)	Vision impaired in both eyes, but optic discs remained pale and had the appearance of optic atrophy.
	12 y<br>F		Tuberculous meningitis, Miliary spread	Prednisone + Thalidomide 5mg/kg/d (3 months)	The pupil reactions remained sluggish to light and the optic discs paler. MRI showed marked improvement.
	2 y F		TBM, tuberculous optochiasmatic arachnoiditi	Prednisone + Thalidomide 5mg/kg/d (3 months)	Vision recovery
M.T.M. Roberts<br>Oct 2003<br><br>*[Reference 40]*	23 y<br>F<br><br>India		Tuberculous meningitis	Prednisolone 30 mg/d + thalidomide 50 mg/2xd (40 months). Thalidomide was reduced 50 mg/d for 3 months	Stable residual field defect of a homonymous hemianopia. Thalidomide adverse effect peripheral sensory neuropathy leading to some loss of fine touch in the fingers
	37 y<br>F		Tb meningitis	Thalidomide 50 mg three times daily + cyclosporin A was introduced temporarily for 2 m.	She dies because of brain vascular injury. No evidence of Tb relapse
Johan F. Schoeman<br>April 2006<br><br>*[Reference 41]*	5 y<br>M		TBM	Prednisone 60 mg/kg/day +<br>thalidomide 3.3 mg/kg/day (4 m)	Full neurological recovery
	6 y<br>M		TBM	Thalidomide 4.5 mg/kg/day (8 months)	Optic disks remained pale. The patient was unable to walk due to spastic diplegia.
	1 y 3 m<br>F		TBM	Prednisone+ Thalidomide 4 mg/kg/day (8 months)	Mild global delay, normal motor function.
S. Keddie<br>June 2018<br><br>*[Reference 42]*	40 y F<br>Indian		Pulmonary TB, TB-IRIS, brain TB abscesses	Thalidomide	Mild memory impairment and mild sensory peripheral neuropathy secondary to thalidomide treatment
	37 y M<br>Senegal		CNS TB	Dexamethasone + thalidomide	Dead
	50 y M<br>Somali		CNS TB	Prednisolone + thalidomide	Wheelchair bound but had regained continence
	41 y F<br>Congo		CNS TB	Dexamethasone 2 mg/week + thalidomide 100 mg BD	Severe cognitive–communicative impairment, walking with frame and assistance
	37 y M<br>Polish		Miliary TB, intracranial tuberculomas	Dexamethasone 16 mg BD + thalidomide 200 mg BD	Upper and lower limb power is improving, severe cognitive impairment

Brunel<br>2012<br><br>*[Reference 43]*	19 y<br>F	HIV	Disseminated TB in context of immunovirological failure	Prednisone 40 mg/kg/day (10 months)+ thalidomide 100 mg/day (14 months)	Improved, no sequelae
Lin Wang<br>Nov 2017<br><br>*[Reference 44]*	2 y 8m M	Crohn		Thalidomide 2.5 mg/kg/day	Remission
	2 y F	Crohn		Thalidomide 2.5 mg/kg/day	No response
	2 y 4 m F	Crohn		Thalidomide 2 mg/kg/day	Remission
	11 y 7 m F	Crohn		Thalidomide 1.8 mg/kg/day	Remission
	12 y 6 m M	Crohn		Thalidomide 2 mg/kg/day	Remission
	12 y 5 m M	Crohn		Thalidomide 1.2 mg/kg/day	Remission
	8 y 6 m M	Crohn		Thalidomide 2 mg/kg per day	Remission
	3 y 8 m F	Crohn		Thalidomide 2.5 mg/kg/day	Remission
	2 y 4 m F	Crohn		Thalidomide 2.2 mg/kg/day	Remission
	13 y 6 m F	Crohn		Thalidomide 1.8 mg/kg/day	Remission
Ronald Van Toorn<br>Mar 2021<br><br>*[Reference 45]*	2 y 2 m<br>F		Tuberculoma multi-loculated	Corticosteroids (21 days) + thalidomide 2.2 mg/kg/day (3 m)	CN VI palsy resolved after 3 wk; full recovery
	7 y F		Tuberculoma multi-loculated	Corticosteroids (7 days)+ thalidomide 3.5 mg/kg/day (3 m)	Cerebellar signs resolved after 10 d; full recovery
	6 y F		TB abscess	Corticosteroids (14 days)+ thalidomide 2.1 mg/kg/day (8 m)	Cerebellar signs resolved after 2 wk; full recovery
	14 y<br>M	HIV	TB abscess	Corticosteroids (12 days)+ thalidomide 2.6 mg/kg/day (86months)	Mild L hemiplegia
	2 y 3 m M		TB abscess (1), tuberculoma (2)	Corticosteroids (11 days)+ thalidomide 3.3 mg/kg/day (4 m)	Hemiplegia resolved after 3 mo
	2 y M		TB abscess (2), tuberculoma (4)	Corticosteroids (28 days)+ thalidomide 3.0 mg/kg/day (9 m)	Dead of brainstem ischemia
	2 y 6 m F		TB abscess	Corticosteroids (7 days)+ thalidomide 5.0 mg/kg/day (6 m)	Full recovery
	4 y F		TB abscess (1), tuberculoma (3)	Corticosteroids (14 days)+ thalidomide 4.1 mg/kg/day (5 m)	Full recovery
	8 m M	HIV-	Tuberculoma multi-loculated	Corticosteroids (28 days)+ thalidomide 2.3 mg/kg/day (1 m)	Full recovery
	12 y M	HIV	TB abscess	Corticosteroids (28 days)+ thalidomide 2.6 mg/kg/day (6 m)	Full recovery
	2 y 2 m M		TB abscess	Corticosteroids (7 days)+ thalidomide 2.6 mg/kg/day (3 m)	Ptosis resolved within 4 wk; full recovery
	8 m M		TB abscess	Corticosteroids (4 weeks)+ thalidomide 3.8 mg/kg/day (5 m)	Independent walking after 3 mo
	10 y F	HIV	Tuberculomas	Corticosteroids (21 days)+ thalidomide 1.8 mg/kg/day (6m)	Full recovery
	11 m F		TB abscess & tuberculoma	Corticosteroids (14 days)+ thalidomide 3.8 mg/kg/day (4 m)	Full recovery
	4 y 6 m F		Tuberculoma	Corticosteroids (5 days)+ thalidomide 2.3 mg/kg/day (5 m)	Full recovery
	5 y 4 m M		Tuberculoma multi-loculated	Corticosteroids (18 days)+ thalidomide 4.0 mg/kg/day (3 m)	Mild L hemiplegia
	5 y M		Optochiasmatic arachnoiditis	Corticosteroids (3 days)+ thalidomide 3.6 mg/kg/day (2 mo)	Normal vision within 2 weeks
	3y F	HIV	Tuberculoma	Corticosteroids (15 days)+ thalidomide 3.7 mg/kg/day (2 m)	Normal vision after 2 weeks
	2 y F	HIV	Tuberculoma	Corticosteroids (12 days)+ thalidomide 2.5 mg/kg/day (1 m)	Asymptomatic no motor deficit
	3 y 8 m F		Tuberculoma multi-loculated	Corticosteroids (7 days)+ thalidomide 4.1 mg/kg/day (1 m)	Asymptomatic no motor deficit
	2 y M		TB abscess (1), tuberculoma (3)	Corticosteroids (7 days)+ thalidomide 5.0 mg/kg/day (1 m)	Asymptomatic no motor deficit
	4 y M		TB abscess	Corticosteroids (6 days)+ thalidomide 2.5 mg/kg/day (4 m)	Hemiplegia resolved within 2 mo
	1 y 6 m M		TB abscess (1), tuberculomas(3)	Corticosteroids (28 days)+ thalidomide 7.1 mg/kg/day (2 m)	R sided weakness resolved within 4 wk
	2 y M		TB abscess	Corticosteroids (8 days)+ thalidomide 6.0 mg/kg/day (2 m)	Surgically drained. Ataxia resolved within 4 wk
	2 y 4 m M		Multiple tuberculomas	Corticosteroids (7 days)+ thalidomide 5.3 mg/kg/day (4 m)	Full recovery
	2 y M		Tuberculoma	Corticosteroids (12 days)+ thalidomide 2.1 mg/kg/day (8 m)	Hemiplegia recovered within 4 wk
	8 y M		TB abscess (1), tuberculoma (2)	Corticosteroids (28 days)+ thalidomide 2.3 mg/kg/day (1 m)	Full recovery
	5 y F	HIV	Tuberculoma multi-loculated	Corticosteroids (28 days)+ thalidomide 3.9 mg/kg/day (2 m)	Full recovery
	1 y 9 m F		TB abscess	Corticosteroids (28 days)+ thalidomide 2.7 mg/kg/day (3 m)	Mild R hemiplegia
	1 y F	HIV	TB abscess (1), tuberculoma (3)	Corticosteroids (5 days)+ thalidomide 7.1 mg/kg/day (4 m)	No further infarcts or enhancement
	12 y F		TB abscess & tuberculomas	Corticosteroids (4 days)+ thalidomide 2.2 mg/kg/day (3 m)	CN III palsy resolved after 1 mo; full recovery
	12 M		Tuberculoma multi-loculated	Corticosteroids (5 days)+ thalidomide 3.3 mg/kg/day (3 m)	Bilateral CN VI palsies resolved after 6 wk; full recovery

	10 y F		Tuberculoma multi-loculated	Corticosteroids (12 days)+ thalidomide 3.2 mg/kg/day (1 mo)	EPC resolved within 2 wk
	5 y 10 m F		TB abscess	Corticosteroids (28 days)+ thalidomide 4.1 mg/kg/day (1 m)	Surgical decompression; full recovery
	8 y M	HIV	Optochiasmatic arachnoiditis	Corticosteroids (6 days)+ thalidomide 2.3 mg/kg/day (1 m)	Full recovery of vision within 4 wk
	1 y 8 m M		Optochiasmatic arachnoiditis	Corticosteroids (6 days)+ thalidomide 3.5 mg/kg/day (2 m)	Full recovery of vision after 3 wk
	5 m, F		Tuberculoma multi-loculated	Corticosteroids (4 weeks)+ thalidomide 3.2 mg/kg/day (3 m)	Ataxia resolved within 4 wk; full recovery
	1 y 5 m M		Tuberculoma multi-loculated	Corticosteroids (4 weeks)+ thalidomide 4.5 mg/kg/day (3 m)	CN VI palsy resolved after 3 wk; mild L hemiplegia
Camille Fourcade<br>Nov 2014<br><br>*[Reference 46]*	51 Y<br>F	HIV	TB-IRIS	Prednisone 40 mg + thalidomide 100 mg (5 months)	Fully recovered
Gori Andrea<br>Aug 2000<br><br>*[Reference 47]*	60 y<br>M	HIV	TB lympadenitis, pulmonary TB	Thalidomide 200 mg/day	Thalidomide provoked nausea and itching.<br>The patient fully recovered
	31 y<br>M	HIV	TB lympadenitis, pulmonary TB	Thalidomide 200 mg/day	Thalidomide adverse effects were increase of transaminases and oedema. fully recovered
	29 y<br>M		Pulmonary TB	Thalidomide 200 mg/day	Thalidomide adverse effects were loss of appetite and itching. The patient fully recovered
	59 y<br>F		Pulmonary TB	Thalidomide 200 mg/day	Thalidomide adverse effects were hypotension and paresthesia. The patient fully recovered
	60 y<br>F		Disseminated	Thalidomide 200 mg/day	Thalidomide adverse effects were constipation and arthralgia. The patient fully recovered
Viel-Thériault Isabelle<br>Nov 2016<br><br>*[Reference 48]*	13 y<br>F<br>Nepal		Pulmonary and disseminated TB. TBM	Dexamethasone 0.4 mg/kg/d + thalidomide 6 mg/kg/d x 2 doses. Then dexamethasone 5 mg/d + thalidomide 2 mg/kg/d. Third cycle: dexamethasone 0.4 mg/kg/d + thalidomide 6 mg/kg/d x 2 doses	subtle memory difficulties and moderate optic neuropathy
Johan F. Schoeman<br>August 2000<br><br>*[Reference 49]*	Total n= 15 patients<br>Median age= 34 months		Stage 2 TBM<br>All patients had hydrocephalus: 5 patients noncommunicating, the others had communicating	Prednisone 4-60 mg/kg/ day (1 month) + Thalidomide was administered in different dosages: 4 patients received 6 mg/kg/day, 4 patients received 12 mg/kg/day and 7 received /24 mg/kg/day (4 weeks)	Two patients had increase of transaminases but restored to normal range after changing the anti-TB therapy.<br>One patient that received 24 mg/kg/day developed measles at week 4 and died of aspiration of gastric contents, pulmonary TB and CNS TB.<br>1 patient developed hypsarrhythmia and infantile spasms with severe developmental delay. 1 had moderate developmental delay.<br>5 patients out of 15 had an improvement of the motor function 6 months after administration of Thalidomide. Four patients who presented with hemiparesis had completely normal motor function on physical examination after therapy.
Ronald van Toorn<br>July 2014<br><br>*[Reference 50]*	10 y<br>F	HIV	TBM	Prednisone was given for the first 4 weeks of therapy 2 mg/kg, maximum 60 mg daily<br>Thalidomide 4 mg/kg/d (2 months)	Full recovery
	8 y M	HIV	TBM	Thalidomide 4.8 mg/kg/d (5 months)	Mild right hemiplegia
	14 y M	HIV	TBM	Thalidomide 4 mg/kg/d (6 months) and then 2 mg/kg/d (4m)	Mild left hemiplegia. Visual impairment
	12 y M	HIV	TBM	Thalidomide 3.3 mg/kg/d (6 months)	Full recovery
	5 y 4 m M		TBM	Thalidomide 3 mg/kg/d (3 months)	Moderate left hemiplegia
	2 y M		TBM	Thalidomide 3.2 mg/kg/d (3 m) and then 5 mg/kg/d (9m)	Died
	2y 2 m F		TBM	Thalidomide 4.3 mg/kg/d (7 months)	Endocrine dysfunction
	4 y F		TBM	Thalidomide 3.2 mg/kg/d (6 months)	Full recovery
	2 y 6 m F		TBM	Thalidomide 4.1 mg/kg/d (5 months)	Full recovery
	2 y 3 m M		TBM	Thalidomide 3.5 mg/kg/d (3 months)	Full recovery

	7 y F		TBM	Thalidomide 5 mg/kg/d (8 months)	Full recovery
	2 y 2 m M		TBM	Thalidomide 3.2 mg/kg/d (3 months)	Full recovery
	6 y F		TBM	Thalidomide 4.1 mg/kg/d (9 months)	Full recovery
	8 m M		TBM	Thalidomide 3.2 mg/kg/d (9 months)	Full recovery
	1 y F		TBM	Thalidomide 4.5 mg/kg/d (2 months)	Full recovery
	4 y F		TBM	Thalidomide 2.2 mg/kg/d (5 months)	Full recovery
Ronald Van Toorn<br>Nov 2012<br><br>*[Reference 51]*	10 y<br>F		TBM	Dexamethasone + thalidomide 2 mg/kg/day	Died
	12 y<br>M	HIV	Brain tuberculomas<br>CNS TB-IRIS	Prednisone + thalidomide 2 mg/kg/day	Stevens Johnson reaction and thalidomide was interrupted
	14 y F	HIV	HIV-related tuberculosis- IRIS	corticosteroid 2 mg/kg/d + thalidomide 2 mg/kg/day	Full recovery Mild left hemiplegia
Aayesha J. Soni<br>Sept 2024<br><br>*[Reference 32]*	41y<br>M		CNS TB	Prednisone 2.5 mg/kg/day + thalidomide 2 mg/kg/day	Multiple infections of his sacral pressure sore and urinary tract following the start of TB treatment
	22y F		Miliary TB, CNS TB	Prednisone 2 mg/kg/day + thalidomide 100 mg/day	Residual right toe weakness and impaired proprioception
	16 y<br>M		TB meningitis<br>Confusion and paraplegia	Prednisone 2 mg/kg/day + thalidomide 100 mg/daily (6 months)	Possible drug-induced neutropenia.<br>Residual lower limb spasticity
